# Factors Influencing Fluoroquinolone Resistance

**DOI:** 10.3201/eid0912.03-168

**Published:** 2003-12

**Authors:** Daniel F. Sahm, Clyde Thornsberry, Mark E. Jones, James A. Karlowsky

**Affiliations:** * Focus Technologies, Herndon, Virginia, USA

**Keywords:** fluoroquinolones, resistance, mutation, *Pseudomonas aeruginosa*, *Streptococcus pneumoniae*

**To the Editor:** Recently, Scheld summarized factors that he considered to have an influence on the efficacy of fluoroquinolones (*1*). In the review, ciprofloxacin was presented as the most active fluoroquinolone against *Pseudomonas aeruginosa* with MICs typically two- to eightfold lower than those for levofloxacin, moxifloxacin, or gatifloxacin. However, because the National Committee for Clinical Laboratory Standards (NCCLS) MIC interpretative breakpoints are fluoroquinolone-specific, percent susceptibility is considered to be a better measure by which to compare fluoroquinolone activities. Our company has conducted annual investigations called TRUST (Tracking Resistance in the United States Today) since 1996. These surveillance studies have consistently shown similar susceptibility rates for levofloxacin (67.7% in 2002) and ciprofloxacin (67.4% in 2002) against *P. aeruginosa* (*2*,*3*). Both agents show higher in vitro activity against *P. aeruginosa* than gatifloxacin and moxifloxacin (*2*–*4*). A critique of antipseudomonal fluoroquinolone activity should also consider peak achievable fluoroquinolone levels at a site of infection, the area under the serum concentration curve in 24 hours (AUC_24h_), and the AUC_24h_/MIC ratio (*5*). At equivalent dosages for nosocomial pneumonia, levofloxacin (750 mg intravenously, once daily) has a threefold higher peak serum level (C_max_) and threefold higher AUC_24h_ than ciprofloxacin (400 mg intravenously, every 8 hours) (package inserts for Levaquin and Cipro). While certain *P. aeruginosa* isolates have lower ciprofloxacin than levofloxacin MICs, the two fluoroquinolones have equivalent activity against *P. aeruginosa* because of their equivalent AUC_24h_ /MIC ratios (*6*). We agree strongly with Scheld’s suggestion that the fluoroquinolone used clinically should be the fluoroquinolone tested by the laboratory and reported; surrogate testing of fluoroquinolones may lead to major errors in reporting, particularly for *Enterobacteriaceae* (*2*,*3*,*7*).

The review also stated that levofloxacin-resistant strains of *P. aeruginosa* emerge at a significantly higher rate than with ciprofloxacin. However, a recent study of *P. aeruginosa* isolated from cystic fibrosis patients reported that fewer resistant mutants were isolated after exposure to levofloxacin (11 mutants) than to ciprofloxacin (28 mutants) (*8*).

With regards to *S. pneumoniae*, the review stated that in vitro studies have demonstrated that ciprofloxacin (1–4 mg/L) and levofloxacin (1–2 mg/L) are not as active as moxifloxacin (0.06–0.25 mg/L) and gatifloxacin (0.5–1 mg/L) against pneumococci. As with *P. aeruginosa*, fluoroquinolone comparisons against *S. pneumoniae* should not be limited to MICs alone because pharmacokinetic and pharmacodynamic characteristics differ for each fluoroquinolone. Pneumococcal time-kill studies with levofloxacin, gatifloxacin, and moxifloxacin in a pharmacodynamic model have demonstrated that these three agents possess equal bactericidal activity and are equally effective in preventing resistance development because the lower in vitro MICs for gatifloxacin and moxifloxacin were offset by the higher serum and tissue levels of levofloxacin (*9*). In the same study, ciprofloxacin did not exhibit rapid killing and selected for resistance faster than the other three agents (*9*). TRUST and other U.S. surveillance studies, using the NCCLS-recommended broth-dilution method, have shown that *S. pneumoniae* remain highly susceptible to levofloxacin with resistance rates in the United States of <1%; the MIC_90_ for levofloxacin in these studies has remained at 1 mg/L from 1997 through 2002 (*10*–*15*). Further, levofloxacin, gatifloxacin, and moxifloxacin are equally effective in rates of clinical cure and microbiologic eradication of pneumococcal respiratory infections (*16*, and FDA website; available from: URL: http://www.fda.gov/cder/foi/nda/99/21061_Tequin_medr_PI and http://www.fda.gov/cder/foi/nda/2001/21277_Avelox_medr_PI)

The review implied that, in general, higher AUC_24h_/MIC ratios were associated with better patient outcomes. For *S. pneumoniae*, several pharmacodynamic studies have demonstrated that a target AUC_24h_/MIC ratio of 30 to 35 for fluoroquinolones is the best correlate for successful bacteriologic eradication, clinical cure, and prevention of emergence of resistance during therapy (*5*,*11*,*21*–*23*). Levofloxacin, gatifloxacin, and moxifloxacin all achieve this AUC_24h_/MIC ratio (*9*). Zhanel et al. demonstrated that AUC_24h_/MIC ratios above the target value of 30 to 35 did not improve bacteriologic eradication or reduce the emergence of resistance (*9*). Moreover, no clinical data support the claim that higher AUC_24h_/MIC ratios correlate with better patient outcomes.

The review discusses the question of whether C-8-methoxyquinolones (moxifloxacin and gatifloxacin) have a lower propensity to select resistant mutants of *S. pneumoniae* compared with levofloxacin. Mutation prevention concentration is a theoretical laboratory concept based on agar dilution methodology, and no published data have shown any clinical correlation between this theory and clinical outcomes. NCCLS does not recommend agar dilution for susceptibility analysis of *S. pneumoniae*. Moreover, the extremely low levels of resistance in *S. pneumoniae* (<1%) after many years of fluoroquinolone use do not support the theory of mutation prevention concentration. The review did not reference an analysis of 16 penicillin-resistant *S. pneumoniae* strains by Kolhepp et al. (*20*). In that broth-dilution study, in vitro resistance developed in a greater proportion of strains exposed to gatifloxacin (11/16) and moxifloxacin (8/16) than to levofloxacin (2/16). Similarly, in a study by Klepser et al. that used an in vitro pharmacodynamic model, levofloxacin was less likely than moxifloxacin to select for resistant isolates of *S. pneumoniae;* moreover, after 24 hours of exposure, levofloxacin MICs remained unchanged while moxifloxacin MICs increased two- to eightfold (*21*).

Levofloxacin, gatifloxacin, and moxifloxacin all have susceptibility rates >99% for *S. pneumoniae* (*22*,*23*). Although resistance is rare, considerable cross-resistance among fluoroquinolones is observed once two or more key mutations (e.g., Ser^79^ in ParC, Ser^81^ in GyrA) are detected (*24*,*25*). Using topoisomerase IV-selecting fluoroquinolones (ciprofloxacin and levofloxacin) in the same patient population as DNA gyrase-selecting fluoroquinolones (gatifloxacin and moxifloxacin) could potentially accelerate the development of double mutants (ParC and GyrA) and clinically important class resistance because selective pressure would be applied to both enzyme targets (*26*).

The review stated that, since 1999, at least 20 case reports of pulmonary infection that did not respond to levofloxacin therapy have been published. This number is remarkably small considering that >250 million patients have been treated with levofloxacin worldwide. A number of the treatment failures cited had documentation of prior ciprofloxacin use and ciprofloxacin failure, and many isolates were not tested for levofloxacin susceptibility before treatment (*27*). We agree with the recommendation in the cited Davidson et al. reference: a patient’s failure to respond to one fluoroquinolone is sufficient reason not to use other fluoroquinolones (*27*). Isolated clinical failures will occur with the use of any antimicrobial agent when treating pneumococcal pneumonia.

The notion that fluoroquinolone therapy can be “targeted” for an indication requires challenge as fluoroquinolone therapy will always result in systemic drug levels. Evidence does not indicate that the use of two fluoroquinolones, such as ciprofloxacin and moxifloxacin, minimizes fluoroquinolone resistance. Targeted fluoroquinolone therapy may in fact have adverse implications for the patient and for overall institutional resistance patterns. For example, the use of ciprofloxacin for urinary tract infections exposes resident streptococci in the respiratory tract to an agent that has demonstrated weaker activity against pneumococci, thus potentially selecting for pneumococcal resistance (*9*). Moreover, 20%-35% of ciprofloxacin is excreted through the intestinal tract (Cipro package isnert), compared to 4% of levofloxacin (Levaquin package insert). Studies have shown that ciprofloxacin displays weaker in vitro activity (lower percentage of isolates susceptible) than levofloxacin for several gram-negative enteric bacteria (*2*,*3*). Stepwise adaptive changes towards fluoroquinolone resistance in enteric bacteria may be selected by fluoroquinolones with weaker in vitro activity and higher levels of exposure in the intestinal tract. Therefore, ciprofloxacin would have a greater potential than levofloxacin for the selection of resistant strains for intestinal gram-negative pathogens. A recent report stated that ciprofloxacin-resistant *Escherichia coli* were isolated from the feces of 48% of patients treated with ciprofloxacin for prostatitis; before ciprofloxacin therapy, only ciprofloxacin-susceptible *E. coli* were isolated from the feces of these patients (*28*). Further, given that 25% of moxifloxacin is excreted through the intestinal tract (Avelox package insert), the use of moxifloxacin for respiratory infections exposes pathogens in the intestinal tract to a fluoroquinolone with higher anaerobic activity against *Bacteroides fragilis* and other intestinal anaerobes than levofloxacin (*29*,*30*). Moxifloxacin has a greater potential than other fluoroquinolones to alter the normal intestinal flora and select for vancomycin-resistant enterococci (*31*) and strains of intestinal gram-negative strains with increased resistance towards fluoroquinolones.

In conclusion, we believe that the data we have briefly presented here supplements the previous discussion by Scheld (*1*) and will help facilitate an improved understanding of the factors influencing the maintenance of fluoroquinolone efficacy.
